# Culturable bacterial diversity and genome-encoded metabolic potential in Al Wahbah Crater’s volcanic soils, Saudi Arabia

**DOI:** 10.3389/fmicb.2026.1867957

**Published:** 2026-07-10

**Authors:** Júnia Schultz, Andrii Romanenko, Alexandre Soares Rosado

**Affiliations:** 1 Biological and Environmental Science and Engineering Division, King Abdullah University of Science and Technology, Thuwal, Saudi Arabia; 2 Center for Nuclear Energy in Agriculture, University of São Paulo, Piracicaba, Brazil

**Keywords:** bacterial strains, enzymes, genomic characterization, natural product biosynthesis, volcanic microbiome

## Abstract

The growing demand for sustainable biotechnological solutions has intensified interest in microorganisms inhabiting understudied and environmentally constrained ecosystems. Volcanic systems create ecological niches shaped by geochemical and physicochemical stressors, yet the culturable bacteria in many remain poorly characterized. Here, we investigated the culturable bacterial fraction in the soils of Al Wahbah Crater (Saudi Arabia), an underexplored volcanic ecosystem, and evaluated its members’ hydrolytic enzyme production and the genome-encoded metabolic potential of the most-promising isolates. Using multiple media and incubation conditions, we isolated bacterial strains from three crater soil types, identifying 65 representative isolates through 16S rRNA gene sequencing. The culture collection was dominated by Bacillota, particularly *Bacillus* spp., reflecting selective pressures typical of mineral-rich, saline soils. All isolates were screened for six hydrolases: cellulase, xylanase, amylase, protease, lipase, and gelatinase. Twenty-three strains exhibited activities for all six, while only one (*Paenibacillus* sp. AWC54) showed no detectable enzymatic activity. Cellulase activity was most prevalent (61/65 isolates), followed by xylanase (59/65), amylase (57/65), protease (48/65), lipase (35/65), and gelatinase (31/65). Five high-performing strains, *Bacillus spizizenii* AWC2, *B. cereus* AWC16, *B. vallismortis* AWC57 and AWC81, and *B. haynesii* AWS14, were selected for whole-genome sequencing and genome mining. Genome annotations revealed diverse carbohydrate-active enzyme repertoires including glycoside hydrolases, glycosyl transferases, and polysaccharide lyases, as well as multiple biosynthetic gene clusters predicted to encode antimicrobial and antifungal metabolites. Together, these findings establish Al Wahbah Crater as a cultivable regional reservoir of metabolically versatile bacteria and provide a curated strain collection and genomic framework for future ecological, evolutionary, and biotechnological investigations of volcanic microbiomes in the Arabian Peninsula.

## 1 Introduction

Volcanic craters represent ecological niches that harbor highly diverse and often uncharacterized microbial communities (Shu and Huang, 2022; Herrera and Cockell, 2007). Microorganisms inhabiting these habitats have evolved under intense selective pressures, providing them with specialized metabolic pathways that support survival under harsh conditions, including high temperatures, extreme pH, and limited nutrient availability (Rothschild and Mancinelli, 2001). Investigating these stress-tolerant microbial assemblages provides critical insights into their ecological roles and adaptive strategies while also uncovering their potential for biotechnological applications, such as enzyme production and bioactive compound synthesis (Contreras et al., 2024; Marzban and Tesei, 2025; Schultz and Rosado, 2020).

Saudi Arabia hosts Al Wahbah Crater, a large volcanic structure located in the western region of the Kingdom (Schultz et al., 2026; Dos Santos et al., 2024). Formed through phreatomagmatic activity, the crater’s environment combines alkaline soils, high salinities, and elevated temperatures, creating a habitat conducive to the persistence of stress-adapted microbial taxa (Dos Santos et al., 2024; Albokari et al., 2018). However, the culturable fraction of Al Wahbah Crater microbiome remains insufficiently documented, limiting our ability to connect environmental selection pressures with microbial phenotypes and identify downstream applications. Cultivation-based approaches remain essential for the isolation and characterization of bacteria with relevant metabolic traits, as they enable the evaluation of these bacteria for potential industrial and biotechnological applications and provide reference strains that can complement culture-independent surveys (Schultz et al., 2023a). Building regional culture collections is particularly valuable in the Arabian Peninsula, where extreme habitats are widespread, but cultivation resources remain comparatively limited.

Hydrolytic enzymes, such as proteases, cellulases, and lipases, are of considerable industrial importance and find applications in sectors ranging from the food and pharmaceutical industries to biofuel and detergent production. Microorganisms inhabiting harsh environments frequently produce enzymes with enhanced stability and activity under extreme conditions, making them particularly valuable for industrial processes (Mesbah, 2022; Littlechild, 2015). However, establishing enzyme robustness requires biochemical characterization under relevant operating conditions; therefore, culture-based screening represents an important first step in prioritizing strains for downstream validation. Beyond enzyme production, the genomic analysis of cultured isolates can reveal their full metabolic potential by identifying genes encoding hydrolytic enzymes and secondary metabolites with antimicrobial or other bioactive properties. Such genomic exploration not only sheds light on the adaptive strategies of stress-tolerant bacteria but also highlights their potential as sources of bioactive metabolites and catalogs their enzyme repertoires for future discovery pipelines (Hernandez-Patlan et al., 2022).

Here we characterize the diversity of culturable bacteria in soil samples from Al Wahbah Crater and bioprospect isolates for hydrolytic enzyme production. Furthermore, genomic analyses were conducted to identify genes associated with enzymatic activity and secondary metabolite biosynthesis, providing insights into the adaptive strategies of these culturable crater-associated bacteria and their potential applications. By integrating cultivation-based and genomic approaches, this work establishes a curated strain resource for an underexplored volcanic ecosystem in the Arabian Peninsula and provides a framework for future ecological, evolutionary, and biotechnological investigations.

## 2 Materials and methods

### 2.1 Study site and sampling strategy

**Figure 1:**
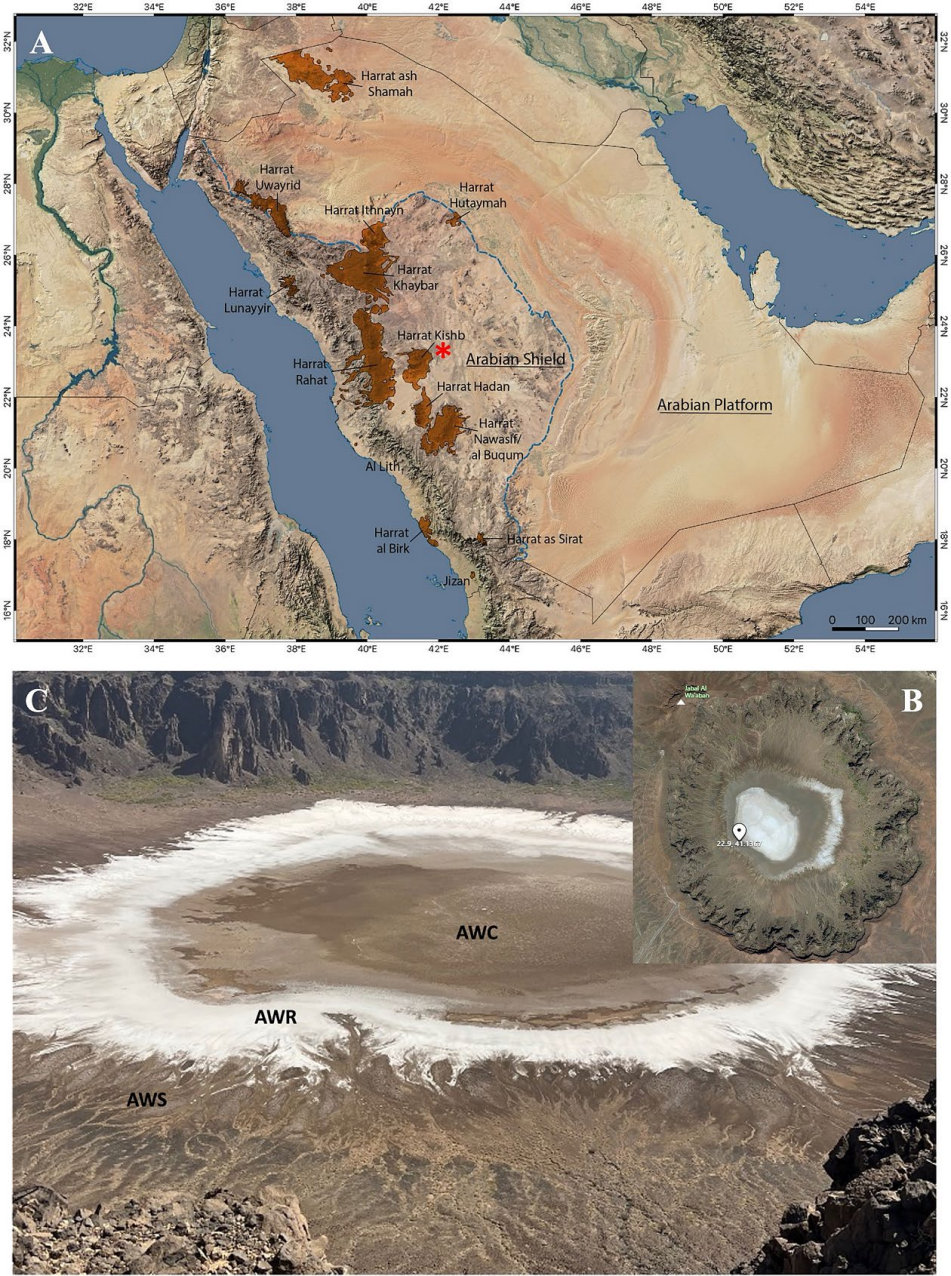
**(A)** A map of Saudi Arabia highlighting the volcanic fields (brown polygons) and the Harrat Kishb (red *), where Al Wahbah Crater is located. Source: Courtesy of Prof. Froukje M. van der Zwan and Dr. Nico Augustin; map created with QGIS using GEBCO terrain data overlaid with image from Google Earth. Harrat and Shield outlines after Roobol et al. (2002). **(B)** A satellite view of Al Wahbah Crater with the coordinates of the sampling point. Source: Image from Bing Maps. Microsoft product screen shot(s) reprinted with permission from Microsoft Corporation. **(C)** A view of the bottom of Al Wahbah Crater with the soil sampling points identified. Source: Photo by Júnia Schultz.

Sampling was performed in Al Wahbah Crater (22° 54’ N and 41° 8′12.12″E), Makkah province, Saudi Arabia, in April 2021 (Figure 1). Soil samples were collected in triplicate from three distinct soil types in the bottom of the crater, totaling 9 soil samples: outer-crater soils (AWS), soils under a salt crust (AWR), and inner-crater, watery soils (clay, AWC). At each sampling point, approximately 500 g of soil from a depth of 0–10 cm was collected aseptically, placed into sterile plastic bags, and stored at 4 °C until laboratory processing. Detailed physicochemical characteristics of the sampling sites were previously described by Dos Santos et al. (2024).

### 2.2 Microbial isolation and culture conditions

To isolate the culturable microbial fraction from the Al Wahbah Crater soils, samples were used as inoculants with five culture media: Reasoner’s 2A agar (R2A; Reasoner and Geldreich, 1985), diluted R2A (1:10), R2A supplemented with 3 and 10% (w/v) NaCl, and lysogeny agar (LB; Bertani, 1951). The objective of using these different culture media was to maximize the recovery of microbial diversity thriving in Al Wahbah Crater by employing nutrient-rich, oligotrophic, and saline cultivation conditions. Firstly, soil samples were homogenized, and 10 g were placed in 90 mL of saline solution (0.85% w/v NaCl) with glass beads and agitated at 120 rpm for 2 h at room temperature. Five tenfold serial dilutions (10^−1^ to 10^−5^) were prepared in the same diluent, and 0.1 mL of each dilution was spread onto the respective agar media. Plates were incubated at 26 °C and 55 °C for up to 72 h, with each dilution and incubation temperature replicated in triplicate.

After incubation, colonies with visually distinct morphologies were isolated, and pure single colonies were transferred to 15 mL conical tubes containing R2A broth medium (Himedia, India) and incubated at 26 °C with agitation (150 rpm) for up to 48 h. Overnight bacterial cultures were aliquoted in triplicate into cryovials containing glycerol (20% v/v) and stored at −80 °C. All strains isolated from Al Wahbah have been deposited in the KAUST Microbial Vault (Supplementary Figure S1).

### 2.3 Genomic DNA extraction, 16S rRNA gene sequence analysis, and taxonomic identification

Prior to DNA extraction, microbial strains were cultured in R2A broth for 48 h at 26 or 55 °C with constant shaking (150 rpm). Genomic DNA was extracted via enzymatic lysis using the Wizard® Genomic DNA Purification Kit (Promega, USA), following the manufacturer’s instructions. Bacterial strains were identified based on an approximately 1,450 bp section of their 16S rRNA gene, which was amplified using the universal primers 27F (5′-AGA-GTT-TGA-TCM-TGG-CTC-AG-3′) and 1492R (5’-TAC-GGY-TAC-CTT-GTT-ACG-ACT-T-3′; Weisburg et al., 1991). The PCR reactions were performed using 50 μL cocktails consisting of 40.8 μL of water, 5 μL of Buffer II, 1 μL of each primer, 0.2 μL of AccuPrime High Fidelity Taq, and 2 μL of genomic DNA in an Applied Biosystems ProFlex PCR system. The amplification protocol included an initial denaturation step at 94 °C for 2 min, followed by 30 cycles of denaturation at 94 °C for 15 s, annealing at 55 °C for 30 s, and extension at 68 °C for 30 s, with a final extension step at 68 °C for 10 min.

Five μL of PCR product was subjected to 1X TBE gel electrophoresis, run at 90 V and 500 A for 45 min in 1% agarose with Sybr Safe stain (Invitrogen, USA). The gels were then visualized using a Gel Doc™ EZ System. Successful PCR products were quantified in a Qubit 4.0 system with the Qubit® dsDNA HS Assay Kit (Life Technologies, USA) and a Nanodrop system. The PCR products from each bacterial strain were sent to Macrogen Inc. (Seoul, South Korea) for Sanger sequencing (chain-termination method) using primers 27F (5′- AGAGTTTGATCCTGGCTCAG-3′) and 1492R (5′- GGTTACCTTGTTACGACTT-3′; Weisburg et al., 1991).

Raw sequences were submitted to Geneious Prime v. 2023^1^ for quality checking and trimming. The trimmed sequences were searched against the National Center for Biotechnology Information (NCBI) nucleotide collection (nr/nt) reference database using the Megablast program. A phylogenetic tree was constructed using the neighbor-joining method with 1,000 bootstrap replicates in the MEGA 11 software and edited in the interactive Tree Of Life (iTOL; Letunic and Bork, 2021).

### 2.4 Bioprospecting for enzymatic activity

The enzymatic activity of 65 bacterial isolates was screened for six industrially relevant hydrolases (amylase, cellulase, gelatinase, lipase, protease, and xylanase). Isolates were selected based on taxonomic identification inferred from their 16S rRNA gene sequences.

Overall, for the screening of amylase, cellulase, lipolytic enzymes, protease, and xylanase, bacterial isolates were reactivated in R2A broth media for up to 24 h at 26 °C and 150 rpm. A 10 μL aliquot of bacteria culture was applied onto plates containing enzyme-specific media, in triplicate. The plates were then incubated for 72 h at 26 °C. Enzyme production was semi-quantitatively estimated using the hydrolysis index, calculated as the ratio of the halo diameter to the colony diameter (Vilela et al., 2021).

Amylase activity screening was performed on a starch agar medium (Himedia, India). The presence of amylase activity was confirmed by the appearance of a clear halo zone around the colonies after staining with Gram’s iodine solution (Yadav, 2018). Protease activity screening was performed on skim milk agar medium (Himedia, India; 0.5% tryptone, 0.3% yeast extract, 1.5% agar, and 25% skim milk), with protease activity confirmed by the appearance of a clear zone around the colonies, indicating degradation of milk casein (Yadav, 2018). For lipolytic activity, nutrient agar medium was supplemented with Tween 80 at 1%, and an opaque halo around the colonies indicated positive lipolytic activity (Yadav, 2018). Additionally, lipolytic activity was tested on Spirit Blue Agar (Sigma-Aldrich, USA; 10 g/L of casein enzymic hydrolysate, 5.0 g/L of yeast extract, 0.15 g/L of spirit blue, and 17.0 g/L of agar), with the lipase substrate prepared according to the manufacturer’s protocol. The formation of a transparent halo around the colony indicated lipolytic activity.

Cellulase activity was detected on a nutrient agar medium containing carboxymethylcellulose (Himedia, India) at 0.2%. Cellulase activity was identified by the appearance of a clear halo around the tested strain after treatment with Gram’s iodine (Johnsen and Krause, 2014). Xylanase activity screening was conducted on a nutrient agar medium containing beechwood xylan at 0.5% (Himedia, India). The presence of xylanase activity was confirmed by the appearance of a clear zone around the tested strain, following staining with Gram’s iodine (Samanta et al., 2011).

Gelatinase activity screening was performed using the nutrient gelatin stab method on nutrient gelatin (Sigma-Aldrich, USA; 120.0 g/L of gelatin, 5.0 g/L of peptone, and 3.0 g/L of meat extract, with a final pH of 6.8). The bacterial inoculum was cultivated for 24 h and then tubes containing nutrient gelatin were stab-inoculated. The tubes were incubated at 26 °C for 72 h. Gelatin normally liquefies at 28 °C and above; thus, to confirm that liquefaction was due to gelatinase activity, the tubes were immersed in an ice bath for 15–30 min, with hydrolyzed gelatin remaining liquid after cold exposure and unhydrolyzed gelatin, as in the uninoculated control medium, remaining solid (Clarke, 1953).

### 2.5 Whole-genome sequencing, taxonomic and functional annotation

Based on the best results of the isolates’ enzymatic activity, five bacterial strains were selected for whole-genome sequencing (WGS) and the deep characterization of their genomic metabolic potential. A 0.1 μg aliquot of genomic DNA from each bacterial isolate was sent to Macrogen Inc. (Seoul, South Korea) for WGS. Paired-end sequencing libraries (2 × 151 bp) with an average insert size of 350 bp were prepared using Illumina TruSeq Nano DNA library preparation kits, following the manufacturer’s protocol. All the samples were sequenced on an Illumina NovaSeq6000 platform, as recommended by the manufacturer, producing a minimum of 10 Gb of data output per sample.

For genome assembly, raw WGS sequences were evaluated using FastQC (Andrews, 2010). *De novo* assembly was performed using the SPAdes (Bankevich et al., 2012), Edena (Hernandez et al., 2008), and Unicycler (Wick et al., 2017) assemblers. The quality of the assembly was checked using QUAST (Quality Assessment Tool for Genome Assemblies; Gurevich et al., 2013), and the best output of the assemblers was used for downstream analyses. Genome completeness and contamination were assessed using CheckM (Parks et al., 2015).

Functional annotation was performed using a variety of tools. General annotation was performed using the RAST (Rapid Annotation using Subsystem Technology) server, which generates whole-genome annotations for prokaryotic species (Overbeek et al., 2014; Aziz et al., 2008). Enzyme annotation was performed using the dbCAN2 meta server (Zhang et al., 2018), and secondary metabolite annotation was performed using antiSMASH (Blin et al., 2021). PHASTER (PHAge Search Tool Enhanced Release) was used for the rapid identification and annotation of prophage sequences within the bacterial genomes (Arndt et al., 2016; Zhou et al., 2011). Default parameters were used for all software unless otherwise specified.

## 3 Results

### 3.1 Cultured microorganisms from Al Wahbah crater

A total of 205 microorganisms presenting different colony morphologies was isolated from samples collected across the three soil types at Al Wahbah Crater. The greatest number of isolates (*n* = 187) was recovered under mesophilic incubation (26 °C), whereas 18 isolates were obtained under thermophilic incubation (55 °C). The highest number of strains was isolated from the AWC samples (*n* = 116), while the lowest was isolated from the AWS samples (*n* = 24). Across media, R2A yielded the highest number of isolates overall (*n* = 72), followed by 3% NaCl R2A (*n* = 64) and LB (*n* = 40), with fewer isolates recovered on 10% NaCl R2A (*n* = 15) and diluted R2A (1:10; *n* = 14; Supplementary Table S1). Isolate recovery also varied by soil type: the highest numbers of microbial strains were isolated from AWC (*n* = 45) and AWS (*n* = 13) soils on R2A medium, whereas most AWR isolates were recovered on 3% NaCl R2A. Out of the 205 recovered microorganisms, 120 were identified as bacterial isolates, of which 86 were isolated from AWC, 26 from AWR, and 8 from AWS (Supplementary Table S1).

### 3.2 Molecular identification of the cultured bacteria

**Figure 2:**
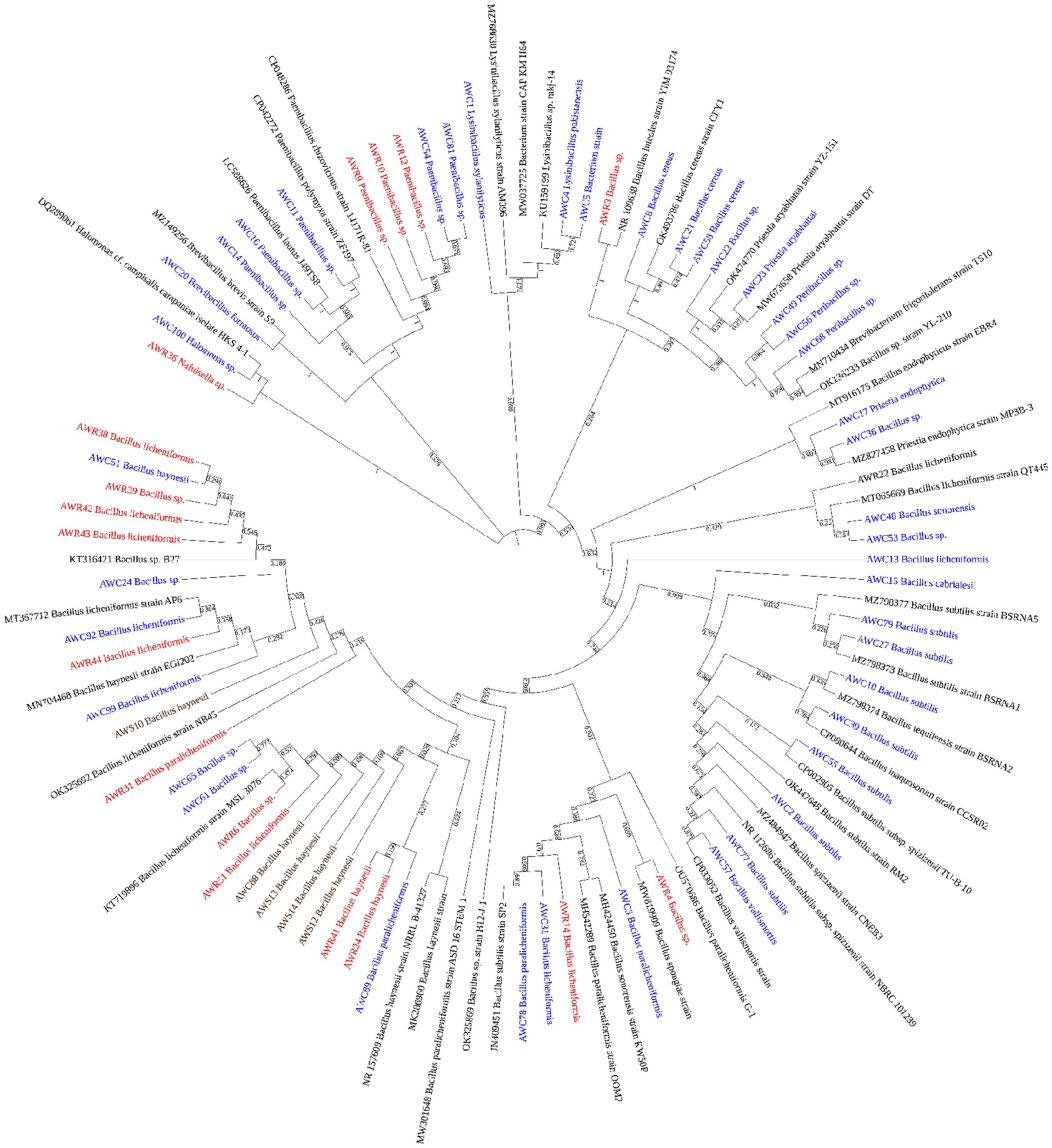
Phylogenetic relationship between isolates identified in Al Wahbah Crater soils. The phylogenetic tree is based on partial sequences of the 16S rRNA gene and shows the relationships between 65 isolates and closely related species from the GenBank database (black). The tree was constructed using the neighbor-joining method in the program MEGA. A bootstrap analysis with 1,000 replicates was performed, and the resulting support values are shown at branch points. Label colors indicate the source of the isolate: strains from AWC soils are marked in blue, strains from AWR soils are marked in red, and strains from AWS soils are marked in brown.

Representing 48 isolates from AWC, 18 from AWR, and 4 from AWS, the majority of the strains were identified as belonging to the phylum Bacillota (97%), while the remaining 3% belonged to Pseudomonadota (1.5%) and Bacteroidota (1.5%). The bacterial isolates that did not belong to Bacillota were identified as *Halomonas* sp. (AWC100) and *Nafulsella* sp. (AWR36). Within AWC samples, the most common taxa were *Bacillus subtilis* (*n* = 7), *Bacillus* sp. (*n* = 6), *Bacillus licheniformis* (*n* = 5), and *Paenibacillus* sp. (*n* = 5). Within AWR samples, *Bacillus licheniformis* was most frequently identified (*n* = 7), followed by *Bacillus* sp. (*n* = 4) and *Paenibacillus* sp. (*n* = 3). All bacterial strains from the AWS samples were identified as *Bacillus haynesii*. While most species were commonly observed across the three soil types, several taxa, including *Bacillus cabrialesii, Bacillus sonorensis, Bacillus vallismortis, Brevibacillus formosus, Lysinibacillus pakistanensis, Lysinibacillus xylanilyticus, Priestia aryabhattai,* and *Priestia endophytica*, were only detected in AWC samples (Supplementary Table S2). The phylogenetic relationships between all isolated bacterial strains and their closest reference sequences in the GenBank database are shown in Figure 2.

### 3.3 Enzymatic activities of the volcanic bacterial isolates

A total of 65 bacterial isolates were screened for six hydrolytic enzyme activities using enzymatic assays, with the results summarized in Table 1. Out of 65 bacterial strains, 23 were positive for the production of all six analyzed enzymes, and these were molecularly identified as members of *Bacillus cabrialesii, B. cereus, B. haynesii, B. licheniformis, B. paralicheniformis, B. sonorensis, Bacillus* sp., *B. subtilis*, *B. vallismortis, Paenibacillus* sp., and *Priestia aryabhattai*. Only one bacterial strain (AWC54), identified as *Paenibacillus* sp., did not test positive for the production of any enzymes. Overall, 61 isolates were positive for cellulase production, 59 for xylanase, 57 for amylase, 48 for protease, 35 for lipase, and 31 for gelatinase. Thus, lipase and gelatinase activities showed the lowest prevalences.

**Table 1:** Hydrolytic enzyme screening results for bacterial isolates from Al Wahbah Crater soils.

Strain	Species	Amylase	Cellulase	Xylanase	Lipase	Protease	Gelatinase
AWC1	*Lysinibacillus xylanilyticus*	Neg	Neg	Neg	Neg	1.33	Neg
**AWC2**	***Bacillus spizizenii***	2.36	2.76	1.69	1.31	1.54	Pos
AWC3	*Bacillus paralicheniformis*	1.43	2.06	2.00	1.23	1.42	Pos
AWC4	*Lysinibacillus pakistanensis*	Neg	2.30	Neg	Neg	1.19	Pos
AWC5	*Bacterium strain*	Neg	2.22	Neg	Neg	1.20	Pos
AWC8	*Bacillus cereus*	1.19	2.21	1.91	1.91	1.84	Pos
AWC10	*Bacillus subtilis*	1.52	2.30	1.27	1.21	1.71	Pos
AWC11	*Paenibacillus* sp.	1.83	2.07	2.56	1.12	2.00	Neg
AWC13	*Bacillus licheniformis*	1.37	2.60	2.46	1.16	1.18	Neg
AWC14	*Paenibacillus* sp.	1.20	1.98	1.74	1.08	Neg	Neg
AWC15	*Bacillus cabrialesii*	1.71	2.38	1.39	1.24	1.33	Pos
**AWC16**	***Paenibacillus/Bacillus* sp.**	1.74	2.30	1.95	1.85	1.85	Pos
AWC17	*Priestia endophytica*	1.63	2.25	1.58	Neg	1.78	Pos
AWC20	*Brevibacillus formosus*	1.17	2.04	1.90	Neg	1.36	Neg
AWC21	*Bacillus cereus*	1.23	2.32	2.01	1.79	1.83	Pos
AWC22	*Bacillus* sp.	1.67	1.82	1.18	Neg	1.61	Pos
AWC23	*Priestia aryabhattai*	1.44	2.01	1.31	1.25	1.93	Pos
AWC24	*Bacillus* sp.	1.26	2.57	2.31	1.29	1.21	Neg
AWC27	*Bacillus subtilis*	1.80	2.01	1.36	1.38	2.00	Pos
AWC31	*Bacillus licheniformis*	1.13	2.11	2.09	1.16	1.20	Neg
AWC36	*Bacillus* sp.	1.37	1.87	1.84	Neg	Neg	Pos
AWC39	*Bacillus subtilis*	1.16	2.61	2.43	1.20	1.90	Pos
AWC48	*Bacillus sonorensis*	1.23	2.63	2.69	1.17	1.25	Pos
AWC49	*Peribacillus* sp.	1.52	1.66	1.30	1.12	1.15	Neg
AWC51	*Bacillus haynesii*	1.27	2.64	1.94	1.39	1.18	Pos
AWC53	*Bacillus* sp.	1.62	2.53	2.61	1.16	1.49	Pos
AWC54	*Paenibacillus* sp.	Neg	Neg	Neg	Neg	Neg	Neg
AWC55	*Bacillus subtilis*	1.41	2.38	1.53	1.32	1.87	Pos
AWC56	*Peribacillus* sp.	1.32	2.18	1.40	1.46	Neg	Pos
**AWC57**	***Bacillus vallismortis***	1.76	2.11	2.84	1.28	1.82	Pos
AWC58	*Bacillus cereus*	1.36	2.64	3.16	Neg	1.38	Neg
AWC61	*Bacillus* sp.	Neg	2.21	1.80	Neg	Neg	Neg
AWC65	*Bacillus* sp.	1.20	2.00	2.01	Neg	Neg	Neg
AWC68	*Peribacillus* sp.	1.43	Neg	Neg	Neg	Neg	Neg
AWC77	*Bacillus subtilis*	1.73	2.28	1.35	1.31	1.85	Pos
AWC78	*Bacillus paralicheniformis*	1.17	5.25	1.83	Neg	Neg	Neg
AWC79	*Bacillus subtilis*	1.67	2.46	1.52	1.09	1.30	Pos
**AWC81**	***Bacillus vallismortis***	1.83	3.47	1.75	1.40	2.33	Neg
AWC88	*Bacillus haynesii*	2.06	2.23	2.00	Neg	Neg	Neg
AWC89	*Bacillus paralicheniformis*	1.57	1.60	1.43	Neg	Neg	Neg
AWC92	*Bacillus licheniformis*	1.46	2.51	2.53	Neg	1.17	Neg
AWC99	*Bacillus licheniformis*	1.31	3.30	1.38	Neg	Neg	Neg
AWC100	*Halomonas* sp.	Neg	1.98	1.64	Neg	1.58	Pos
AWR3	*Bacillus* sp.	Neg	2.40	1.65	Neg	Neg	Neg
AWR4	*Bacillus* sp.	1.29	2.12	1.83	1.19	1.20	Neg
AWR6	*Bacillus* sp.	1.33	2.13	1.91	Neg	1.18	Neg
AWR9	*Paenibacillus* sp.	1.83	2.44	Neg	Neg	2.10	Neg
AWR10	*Paenibacillus* sp.	1.57	Neg	2.22	Neg	Neg	Neg
AWR12	*Paenibacillus* sp.	1.18	2.11	2.02	Neg	1.57	Neg
AWR14	*Bacillus licheniformis*	1.69	2.08	1.91	1.33	1.31	Neg
AWR22	*Bacillus licheniformis*	Neg	1.41	1.34	Neg	Neg	Neg
AWR24	*Bacillus haynesii*	1.71	1.96	2.55	Neg	Neg	Neg
AWR31	*Bacillus paralicheniformis*	1.19	1.88	1.75	Neg	1.25	Neg
AWR36	*Nafulsella* sp.	1.57	4.00	1.89	Neg	Neg	Neg
AWR38	*Bacillus licheniformis*	1.29	2.46	2.59	Neg	1.21	Pos
AWR39	*Bacillus* sp.	2.26	2.36	2.79	1.14	1.29	Neg
AWR41	*Bacillus haynesii*	1.43	2.26	1.98	Neg	1.15	Neg
AWR42	*Bacillus licheniformis*	1.20	2.45	2.38	1.16	1.27	Pos
AWR43	*Bacillus licheniformis*	1.10	2.49	2.89	1.26	1.18	Pos
AWR44	*Bacillus licheniformis*	1.63	1.42	1.97	1.23	1.24	Pos
AWR51	*Bacillus licheniformis*	1.60	2.03	1.91	1.17	1.32	Pos
AWS10	*Bacillus haynesii*	1.38	1.81	1.64	Neg	Neg	Neg
AWS12	*Bacillus haynesii*	1.38	2.85	2.54	1.13	1.80	Neg
AWS13	*Bacillus haynesii*	1.33	2.89	2.43	1.17	1.87	Pos
**AWS14**	***Bacillus haynesii***	1.36	2.63	2.34	1.22	1.90	Pos

Green: Positive result of enzymatic assay. Yellow: Negative result of enzymatic assay. Strains in bold are the bacterial isolates selected for the WGS and genomic analyses.

The five bacterial strains that presented the best results in the enzymatic assays were selected for WGS: AWC2, AWC16, AWC57, AWC81 e AWS14. Among these, AWC81 showed the highest hydrolysis index values for most of the tested activities. Four of the five strains were isolated from the AWC samples, and one was isolated from the AWS samples. Collectively, these patterns indicate widespread hydrolytic potential among the culturable bacterial fraction in Al Wahbah Crater soils.

### 3.4 Genome features of the enzyme-producing bacteria

Whole-genome sequencing was performed for the five selected bacterial isolates that presented the best results in the enzymatic screening. In total, 398,434,740 paired-end reads were generated, and each sample had at least 10 Gb of output. The genome sizes for *Bacillus* strains ranged from 4.2 Mb to 6.2 Mb. The GC contents for strains AWC2, AWC57, and AWC81 were similar (43.7, 43.9, and 43.8%, respectively), while AWS14 had a slightly higher GC content (45.7%), and AWC16 had a lower GC content (35.1%). The number of coding sequences ranged from 4,852 (AWS14) to 7,577 (AWC16). Genome completeness estimates varied across assemblies, reflecting differences in assembly contiguity and/or lineage-specific marker representation. Genome features and relatedness indices are presented in the Table 2.

**Table 2:** Genomic features of the five selected enzyme-producing bacteria from Al Wahbah Crater.

Strain	Species	Genome size (bp)	GC (%)	Coding sequences	RNA	Assembler	Contigs	Coverage	Completeness (%)	Genome accession number	Closest type strain	ANI (%)
AWC2	*Bacillus spizizenii*	4,440,176	43.7	5,475	127	SPAdes	27	5,435X	98.6	JAKYKB000000000	GCA_000227465.1	99.34
AWC16	*Bacillus cereus*	6,239,797	35.1	7,577	153	SPAdes	84	3,554X	74.16	JAKYKC000000000	GCA_006094295.1	98.46
AWC57	*Bacillus vallismortis*	4,293,077	43.9	5,773	114	SPAdes	37	5,475X	94.66	JAKYKD000000000	GCA_004116955.1	99.05
AWC81	*Bacillus vallismortis*	4,483,582	43.8	5,981	107	SPAdes	52	5,750X	55.91	JAKYKE000000000	GCA_004116955.1	99.06
AWS14	*Bacillus haynesii*	4,459,558	45.7	4,852	110	SPAdes	27	5,546X	98.5	JAKYKF000000000	GCA_001969855.1	99.44

### 3.5 Functional insights into Al Wahbah crater’s bacteria

General functional annotations were performed using RAST for the five selected bacterial strains (Table 3). Coding sequences (CDSs) were observed in 27 SEED subcategories. The general genome annotations of strains AWC2, AWC16, AWC57, AWC81, and AWS14 identified a total of 336, 353, 342, 338, and 342 subsystems, respectively. Out of all subsystems, the highest number of CDS were found in the subcategory “amino acids and derivatives,” followed by “carbohydrates,” and then “protein metabolism.” Comparatively, strain AWC16 showed the highest number of CDS in 17 functional categories, followed by AWC81 (in six categories), AWC2 (in three categories), and AWC57 (in one category), whereas AWS14 showed lower CDS counts across most categories.

**Table 3:** General functional annotation of the selected enzyme-producing bacteria isolated from Al Wahbah Crater.

Subsystem	AWC2 *Bacillus spizizenii*	AWC16 *Bacillus cereus*	AWC57 *Bacillus vallismortis*	AWC81 *Bacillus vallismortis*	AWS14 *Bacillus haynesii*
Amino acids and derivatives	402	**489**	423	418	357
Carbohydrates	356	**449**	379	384	354
Cell division and cell cycle	4	**8**	5	5	4
Cell wall and capsule	113	100	**121**	**121**	64
Cofactors, vitamins, prosthetic groups, pigments	180	**224**	233	216	152
DNA metabolism	81	**124**	100	99	68
Dormancy and sporulation	125	**126**	116	140	102
Fatty acids, lipids, and isoprenoids	70	**115**	105	58	47
Iron acquisition and metabolism	39	**97**	41	44	45
Membrane transport	**68**	47	67	66	44
Metabolism of aromatic compounds	13	**22**	17	14	14
Miscellaneous	**38**	32	31	32	29
Motility and chemotaxis	74	16	87	**89**	43
Nitrogen metabolism	**31**	21	30	28	22
Nucleosides and nucleotides	125	**155**	130	145	106
Phages, prophages, transposable elements, and plasmids	5	**30**	5	7	8
Phosphorus metabolism	19	**25**	18	22	11
Photosynthesis	0	0	0	0	0
Potassium metabolism	3	**12**	4	3	2
Protein metabolism	259	224	254	**262**	197
Regulation and cell signaling	32	**37**	34	33	24
Respiration	51	**98**	58	53	44
RNA metabolism	71	81	75	**89**	58
Secondary metabolism	8	**11**	8	9	9
Stress response	57	51	56	**69**	42
Sulрhur metabolism	9	6	9	**11**	7
Virulence, disease and defense	55	**85**	59	58	44

Bold = highest number in each subsystem.

We further examined two SEED categories relevant to environmental persistence and industrial application potential: predicted genes related to stress response and secondary metabolism (Table 4). All strain harbored multiple genes associated with a variety of stress-response functions, including those related to osmotic, oxidative, and periplasmic stressors. The diversity of secondary metabolites predicted by RAST was limited and included thiazole/oxazole-modified microcin (TOMM) synthesis, alkyl pyrone synthase, and auxin biosynthesis. Genes encoding auxin biosynthesis enzymes were found in all strains, while TOMM synthesis genes were only detected in strains AWC16 and AWS14. Regarding stress responses, the highest number of annotated genes from the genomes was those related to choline and betaine uptake and betaine biosynthesis, consistent with osmotic stress mitigation (23 in AWC81, 20 in AWC2, 16 in AWC57, 15 in AWC16, and 11 in AWS14). Genes associated with oxidative stress were present in similar numbers across all strains (range 10–14). The general stress sigma factor SigB regulon was also represented across all genomes, with higher counts (11–13) in AWC2, AWC57, and AWC81.

**Table 4:** Annotation of genes related to stress responses and secondary metabolites in the genomes of the five selected Al Wahbah Crater bacterial strains.

Stress response	AWC2 *Bacillus spizizenii*	AWC16 *Bacillus cereus*	AWC57 *Bacillus vallismortis*	AWC81 *Bacillus vallismortis*	AWS14 *Bacillus haynesii*
Osmotic stress					
Osmoregulation	1	2	1	1	1
Choline and betaine uptake and betaine biosynthesis	20	15	16	23	11
Oxidative stress					
Protection from reactive oxygen species	3	4	2	4	2
Oxidative stress	12	10	10	12	14
Glutathione: non-redox reactions	1	2			
CoA disulfide thiol-disulfide redox system		4	2		
Glutathione: redox cycle	1	1	1	2	1
glutaredoxins			1	2	
Glutathionylspermidine and trypanothione		1			
Detoxification	4	10	4	6	8
No subcategory					
Flavohemoglobin			1	1	
SigmaB stress response regulation	11	4	12	13	6
Dimethylarginine metabolism	2	2	2	2	2
Bacterial hemoglobins	2		3	2	1
Hfl operon	1	3	1	2	1
Commensurate regulon activation			1		
Carbon starvation	2	1	2	4	2
Periplasmic stress					
Periplasmic stress response	2	2	1	1	1
Secondary metabolites					
Thiazole- oxazole-modified microcin (TOMM) synthesis		4			3
Alkyl pyrone synthase	3		3	4	2
Auxin biosynthesis	5	7	5	5	4

Color gradient: increasing from white to red according to the number of genes annotated in each category and bacterial strain.

The repertoire of carbohydrate-active enzymes (CAZymes) was predicted using the dbCAN2 pipeline, integrating the HMMER, DIAMOND, and eCAMI platforms. The analysis predicted enzymes spanning multiple Enzyme Commission (EC) classes, including transferases, hydrolases, lyases, and isomerases (Supplementary Table S3). Carbohydrate active enzymes were divided into five main classes, including glycoside hydrolases (GHs), glycosyl transferases (GTs), polysaccharide lyases (PLs), carbohydrate esterases (CEs), and auxiliary activities (AAs), and were further divided into families and subfamilies based on the monospecificity of the substrate and coding sequence.

In the CAzy annotations, prediction of GHs, GTs, and PLs were observed. In total, strain AWC16 exhibited the highest number of CAZyme-coding genes (*n* = 51), followed by AWC2 (*n* = 44), AWC57 (*n* = 36), AWC81 (*n* = 36), and then AWS14 (*n* = 27). The highest number of predicted hydrolase-related genes was found in AWC16 (*n* = 41), while the lowest was found in AWS14 (*n* = 21). Consistent with our plate-based screening, all sequenced strains encoded enzymes corresponding to the assayed activities, including amylase (EC 3.2.1.1), endo-*β*-1,4-glucanase/cellulase (EC 3.2.1.4), and endo-β-1,4-xylanase (EC 3.2.1.8). Additional predicted activities included maltogenic *α*-amylase (EC 3.2.1.133; in AWC2, AWC16, and AWS14), xylan 1,4-β-xylosidase (EC 3.2.1.37; in all strains except AWS14), and α-xylosidase (EC 3.2.1.177; in AWC16). Strain AWC16 also uniquely encoded a predicted cellobiohydrolase (EC 3.2.1.91). Overall, most predicted CAZymes were shared across genomes, with a smaller subset of strain-specific functions. Strain AWC16 contained the largest number of unique predicted CAZymes, including primeverose-producing oligo-xyloglucan hydrolase and reducing end-acting cellobiohydrolase, whereas feruloyl esterase was only detected in AWC2 and isoamylase only in AWS14.

**Figure 3:**
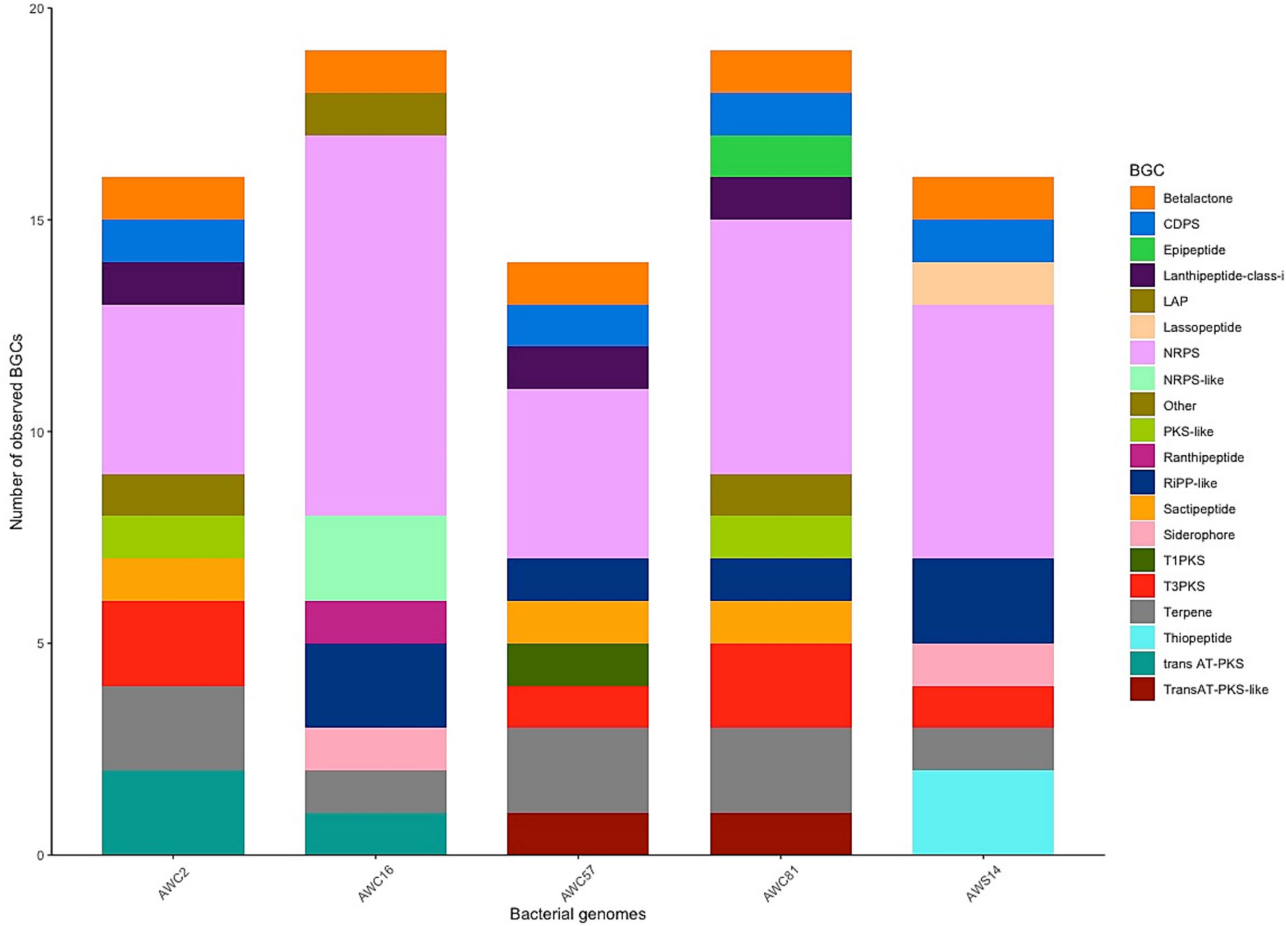
Distribution of biosynthetic gene clusters (BGCs) in the genomes of the five selected enzyme-producing bacteria from Al Wahbah Crater.

In addition to CAZymes, biosynthetic gene clusters (BGCs) were predicted using antiSMASH (Figure 3; Supplementary Table S4). The most common BGC class across the five genomes was non-ribosomal peptide synthetases (NRPS), with nine NRPS clusters in AWC81, six in AWC2 and AWC16, and four in AWC57 and AWS14. Terpene and type III polyketide synthase (T3PKS) clusters were also recurrent, as were several RiPP-like and other predicted cluster classes. Multiple BGCs showed high similarities to previously characterized clusters, including those related to surfactin, fengycin/ plipastatin-family lipopeptides, bacillaene, bacitracin, and siderophores such as bacillibactin (Supplementary Table S4). Strain-specific profiles were also observed: AWC81 harbored predicted clusters with similarities to bacilysin, entianin, and plipastatin; AWC2 harbored a cluster similar to mycosubtilin; AWC57 harbored a cluster similar to zwittermicin A; and AWS14 harbored clusters related to lichenysin.

Additionally, 23 prophage sequences were detected across the five genomes, of which the majority were incomplete, though four intact prophage regions were identified (two in AWS14 and one each in AWC16 and AWC81). The intact prophage regions showed the closest similarities to phages associated with *Brevibacillus* and *Bacillus* (Supplementary Table S5).

## 4 Discussion

This study focused on the recovery and characterization of the culturable bacterial fraction inhabiting the volcanic soils of Al Wahbah Crater. Using culture-dependent approaches, we identified representatives of three bacterial phyla: Bacillota, Pseudomonadota, and Bacteroidota. Compared with previous culture-independent studies based on 16S rRNA amplicon sequencing, which revealed more diverse microbial assemblages including members of Planctomycetes, Chloroflexi, Actinobacteria, and Euryarchaeota (Eida et al., 2018; Albokari et al., 2018), our findings highlight the selectivity of cultivation-based methods. In particular, spore-forming and fast-growing Bacillota, especially *Bacillus* spp., which are known for their ecological resilience in environmentally constrained systems, were favored in the microbial culturing. The predominance of spore-forming Bacillota in Al Wahbah Crater soils is consistent with the findings of dos Santos, who, using saline- and alkaline-enriched culture media, also predominantly recovered Bacillus-related taxa from this volcanic environment.

Bacillota frequently persist in nutrient-poor or disturbed soils due to their capacity for dormancy, metabolic versatility, and rapid physiological reactivation when conditions become favorable. Comparable patterns of *Bacillus* enrichment and functional robustness have been reported in other environments experiencing strong physicochemical constraints, including nutrient-impoverished tropical soils (Huang et al., 2024). Although culture-dependent approaches remain indispensable for obtaining live isolates and experimentally validating microbial functions, they capture only a subset of the environmental microbiome. In contrast, culture-independent approaches provide a broader view of microbial community diversity but do not readily enable downstream physiological or biotechnological characterization (Epstein, 2013; Schultz et al., 2023b). Together, these complementary strategies are essential for advancing the ecological and functional understanding of volcanic microbiomes.

The hydrolytic enzymes produced by the Al Wahbah crater’s bacteria have diverse industrial applications. We detected cellulases, proteases, amylases, lipases, and xylanases, consistent with observations in *Bacillus* species from other extreme environments (Foronda et al., 2025; Priyadarshini and Ray, 2019). These enzymes are relevant to the detergent, food, pharmaceutical, and biofuel industries. Although their enzymatic robustness under extreme physicochemical conditions will require further biochemical validation, the widespread hydrolytic phenotypes observed here provide a basis for prioritizing these strains for downstream characterization.

For example, extremophilic cellulases from halophilic, thermophilic, and psychrophilic *Bacillus* species have been shown to exhibit high activity, particularly those from thermophilic strains. Similarly, *Bacillus*-derived amylases are active in neutral to alkaline soils across a variety of regions (Priyadarshini and Ray, 2019), while thermostable lipases have been reported in Bacillus species isolated from volcanic environments in Chile (Foronda et al., 2025). Xylanases hydrolyze xylan, a major component of the plant cell walls, and enzymes such as endo-1,4-*β*-xylanase (EC 3.2.1.8) and β-xylosidase (EC 3.2.1.37), both predicted in the genomes of strains AWC2, AWC16, AWC57, and AWC81, are essential for complete xylan hydrolysis. Proteases are widely used in detergent, food, pharmaceutical and agricultural applications. Studies on *Bacillus*-derived proteases with enhanced thermal and alkaline stability have demonstrated considerable industrial potential (Karray et al., 2021). Notably, microbial proteases may also inactivate viruses through protein capsid degradation, presenting applications in wastewater treatment (Majchrzak and Poręba, 2022). Lipases, which catalyze lipid hydrolysis across a broad range of temperatures and pH values, are industrially valuable when thermostable above 70 °C and are relevant to food, pharmaceutical, detergent, chemical, and biofuel production (Bora and Bora, 2012). Gelatinases, a subgroup of proteases capable of degrading gelatin, casein, and collagen, also possess relevant biomedical, chemical, and food industry applications (Pathak and Rathod, 2017). The congruence between plate-based enzymatic assays and genome-predicted enzymatic repertoires reinforces the functional and biotechnological potential of these isolates.

The metabolic capabilities observed among the isolated strains also appeared to be closely associated with the physicochemical characteristics of the sampled soils. The predominance of Bacillota, particularly *Bacillus* spp., across the crater soils is consistent with the strong selective pressures imposed by the alkaline, saline, and nutrient-limited conditions characteristic of this volcanic environment. Such conditions likely favor stress-tolerant and metabolically versatile microorganisms capable of surviving environmental fluctuations through dormancy, sporulation, and the production of extracellular hydrolytic enzymes. Notably, the AWC soils yielded the highest number and diversity of isolates, including several strains displaying broad hydrolytic capabilities and enriched repertoires of carbohydrate-active enzymes. The wetter and clay-rich nature of these inner-crater soils may promote greater organic matter retention and microhabitat heterogeneity, thereby supporting microbial diversification and metabolic specialization. In contrast, the AWS and AWR soils, which are likely subjected to stronger salinity and desiccation pressures, were dominated by fewer taxa, particularly *Bacillus haynesii* and related *Bacillus* species, suggesting stronger environmental filtering. Furthermore, these observations indicate that local physicochemical heterogeneity within the crater likely shapes both the taxonomic composition and the functional potential of the culturable bacterial community.

Insights into the metabolic potentials of Al Wahbah Crater’s bacteria were further expanded through comprehensive genomic analyses. Although some assemblies exhibited moderate completeness, core metabolic and biosynthetic pathways were consistently recovered, supporting the functional inferences drawn from the genomic data. *Bacillus* species possess genes encoding a wide array of enzymes, enabling potential versatile metabolic capabilities. For instance, genomic analysis of *B. subtilis* revealed more than 200 carbohydrate-active enzymes, including 65 glycoside hydrolases and 46 glycosyl transferases, highlighting their capacity to utilize diverse carbon sources (Jayakumar et al., 2021). Similarly, the genomes of Al Wahbah Crater isolates exhibited substantial CAZyme diversity, particularly within glycoside hydrolase families, suggesting important ecological roles in organic matter turnover turnover and nutrient cycling within the crater soils.

High secondary metabolite diversities and stress-adaptation traits are not restricted to volcanic systems and have also been widely reported in other environmentally constrained habitats, including mangrove rhizospheres, where cultured isolates displayed both plant growth-promoting and hydrocarbon-degrading capacities (do Carmo et al., 2011). Many of these metabolites predicted in the present study, including as butirosin A/B and zwittermicin A, are active against Gram-positive and Gram-negative bacteria. Bacillaene functions as a polyene antibiotic, whereas fengycin exhibits antifungal activity against filamentous fungi and plant pathogens. Plipastatin is another antimicrobial compound with applications in plant protection (Fazle Rabbee and Baek, 2020). The occurrence of similar secondary metabolites in genomes of *Bacillus* species isolated from both extreme and non-extreme environments suggests that these biosynthetic capabilities are evolutionarily conserved within the genus rather than solely being stress-induced adaptations. For example, *B. halotolerans* from the Qinghai-Tibet Plateau was predicted to produce fengycin, surfactin, and bacillibactin, an iron-chelating agent, while a plant-associated *B. subtilis* strain contained 17 biosynthetic clusters coding for fengycin, surfactin, and bacteriocins (Wu et al., 2021; Jayakumar et al., 2021). Such metabolites may contribute to ecological competitiveness in mineral-rich and saline crater soils through antimicrobial activity, siderophore-mediated iron acquisition, and niche exclusion.

Mobile genetic elements, including insertion sequences and prophages, may further contribute to bacterial adaptability by promoting genomic plasticity and horizontal gene transfer of ecologically advantageous traits, such as antimicrobial production, virulence-associated factors, and resistance to environmental stressors (Vandecraen et al., 2017). The coexistence of stress-response genes, byosinthetic gene clusters, and mobile genetic elements in the genomes of Al Wahbah Crater bacteria suggests a dynamic genomic architecture that may facilitate persistence in the crater’s environmentally fluctuating volcanic conditions.

It is important to acknowledge that the present study is subject to limitations that reflect intrinsic bottlenecks of microbiome culturing approaches. As previously mentioned, the cultivation-based strategy employed here captures only a fraction of the microbial diversity present in Al Wahbah Crater soils; many slow-growing, or yet-uncultivable taxa likely remained undetected. These biases are well-recognized constraints in microbial ecology and highlight the persistent gap between environmental microbial diversity and the fraction currently accessible through laboratory cultivation. Future studies integrating cultivation-independent approaches, including metagenomics and metatranscriptomics, together with expanded cultivation strategies and alternative media formulations, may provide a more comprehensive understanding of the Al Wahbah Crater microbiome and its functional diversity. Additionally, the enzyme screening assays performed in this study relied on halo-formation methodologies, which provide qualitative**/**semi-quantitative indications of hydrolytic activity. Halo size may be influenced by factors unrelated to enzyme production itself, including substrate diffusion, colony morphology, microbial growth rate, and medium composition. Therefore, the observed hydrolysis indices should be interpreted as preliminary indicators of enzymatic potential rather than definitive measures of catalytic efficiency or industrial applicability. Further biochemical characterization, including enzyme purification, kinetic analyses, substrate specificity testing, and stability assessments under different physicochemical conditions, will be necessary to validate the biotechnological potential of the identified strains and their predicted enzymes.

## 5 Conclusion

This study provides a cultivation-based characterization of bacteria inhabiting the volcanic soils of Al Wahbah Crater, Saudi Arabia, integrating bacterial isolation, enzymatic screening, and genome-informed functional analyses. The culturable bacterial fraction was dominated by members of Bacillota, particularly Bacillus-related taxa, reflecting both the selective pressures imposed by the crater’s saline and environmentally constrained conditions and the inherent selectivity of cultivation-based approaches. Despite these limitations, the isolated strains exhibited diverse hydrolytic capabilities, with cellulase, amylase, and xylanase activities being particularly widespread among the recovered bacteria.

Whole-genome sequencing of five selected Bacillus strains revealed diverse repertoires of carbohydrate-active enzymes and biosynthetic gene clusters associated with potentially bioactive metabolites, supporting the metabolic versatility of these microorganisms. Although the predicted enzymatic and biosynthetic functions require additional biochemical and functional validation, the integration of phenotypic assays and genome-based analyses provides important insights into the ecological adaptation and potential biotechnological relevance of culturable bacteria from this underexplored volcanic ecosystem in Saudi Arabia.

Overall, this work expands the current understanding of microbial life in Al Wahbah Crater and establishes a curated regional strain collection that may serve as a valuable resource for future ecological, genomic, and applied microbiology studies. Future investigations combining cultivation-independent methods with advanced biochemical characterization will be essential to further explore the diversity, ecological functions, and applied potential of microorganisms inhabiting volcanic environments in the Arabian Peninsula.

## Data Availability

All Sanger sequences were deposited in the GenBank database (NCBI Sequence Read Archive) and are available as accession numbers OM472023–OM472088 under BioProject accession number PRJNA811115. The genome shotgun projects for strains AWC2, AWC16, AWC57, AWC81, and AWS14 have been deposited at GenBank/NCBI under accession numbers JAKYKC000000000, JAKYKB000000000, JAKYKD000000000, JAKYKE000000000, JAKYKF000000000, respectively, and are available under BioProject accession number PRJNA811115.

## Footnotes

1
www.geneious.com
