# Supplementary material for: Culturable bacterial diversity and genome-encoded metabolic potential in Al Wahbah Crater’s volcanic soils, Saudi Arabia
Source: Front Microbiol. 2026 Jul 10;17:1867957. doi: 10.3389/fmicb.2026.1867957 (PMC13396007; doi:10.3389/fmicb.2026.1867957)
Supplement: Supplementary file 1 [file Supplementary_file_1.DOCX]

Supplementary Material

# Supplementary Figures and Tables

## Supplementary Figure

**
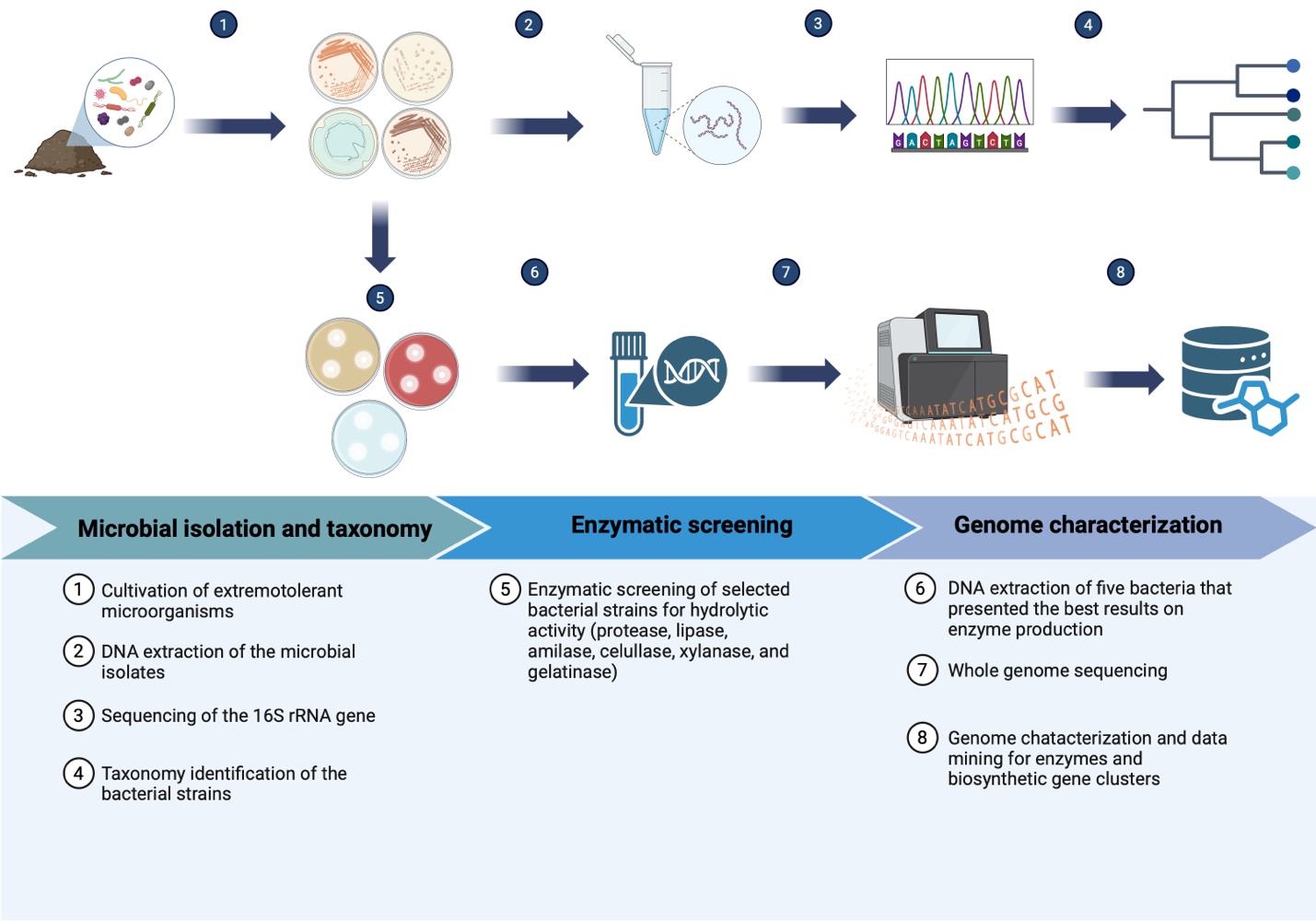
**

**Supplementary Figure 1.** Flowchart summarizing the experimental process. Soil samples were also subjected to DNA extraction for culture-independent analysis. Bacterial isolates were obtained from samples from three soil types and screened for enzymes with potential industrial applications and identified by rRNA 16S gene sequencing (Figure generated in Biorender).

## Supplementary Tables

**Supplementary Table 1.** Number of cultured microbial strains from the Al Wahbah Crater soil samples.

|  | **Soil (AWS)** | **Soil with salt crust (AWR)** | **Clay (AWC)** |
| --- | --- | --- | --- |
| **By temperature** |  |  |  |
| 25°C | 14 | 59 | 114 |
| 55°C | 10 | 6 | 2 |
| **By media** |  |  |  |
| LB | 1 | 13 | 26 |
| R2A | 13 | 14 | 45 |
| dR2A 1:10 | 0 | 1 | 13 |
| R2A 3% NaCl | 10 | 29 | 25 |
| R2A 10% NaCl | 0 | 8 | 7 |
| **Total isolates** | 24 | 65 | 116 |
| Bacteria | 8 | 26 | 86 |
| Other | 16 | 39 | 30 |

**Supplementary Table 2.** Search results for the identification of bacterial strains isolated in this study, showing the reference species and similarity statistics.

| **Strain** | **Temperature of incubation** | **Culture medium for isolation** | **Genbank accession number** | **Reference species (Genbank/NCBI)** | **Similarity (%)** | **Number of bases** | **Reference accession number** |
| --- | --- | --- | --- | --- | --- | --- | --- |
| AWC1 | 26°C | R2A | OM472023 | *Lysinibacillus xylanilyticus* | 99.8 | 838 | MZ950810 |
| AWC2 | 26°C | R2A | OM472024 | *Bacillus subtilis* | 100 | 1373 | OK618379 |
| AWC3 | 26°C | R2A | OM472025 | *Bacillus paralicheniformis* | 99.6 | 1346 | MG651169 |
| AWC4 | 26°C | R2A | OM472026 | *Lysinibacillus pakistanensis* | 98.35 | 1387 | NR_113166.1 |
| AWC5 | 26°C | R2A | OM472027 | *Bacterium strain* | 99.8 | 1349 | MW037796 |
| AWC8 | 26°C | R2A | OM472028 | *Bacillus cereus* | 99.8 | 1361 | OK493787 |
| AWC10 | 26°C | R2A | OM472029 | *Bacillus subtilis* | 100 | 1397 | NR_104873 |
| AWC11 | 26°C | R2A | OM472030 | *Paenibacillus* sp*.* | 99.8 | 1382 | MH842737 |
| AWC13 | 26°C | R2A | OM472031 | *Bacillus licheniformis* | 99.9 | 1112 | MW007811 |
| AWC14 | 26°C | R2A | OM472032 | *Paenibacillus* sp*.* | 99.9 | 1415 | MK139536 |
| AWC15 | 26°C | R2A | OM472033 | *Bacillus cabrialesii* | 99.9 | 1357 | OK189549 |
| AWC16 | 26°C | R2A | OM472034 | *Bacillus* sp. | 99.9 | 1394 | MG594636 |
| AWC17 | 26°C | R2A | OM472035 | *Priestia endophytica* | 100 | 1380 | MZ571869 |
| AWC20 | 26°C | R2A | OM472036 | *Brevibacillus formosus* | 100 | 1406 | OK087333 |
| AWC21 | 26°C | R2A | OM472037 | *Bacillus cereus* | 100 | 1383 | OK493787 |
| AWC22 | 26°C | R2A | OM472038 | *Bacillus* sp. | 100 | 1416 | OK583858 |
| AWC23 | 26°C | R2A | OM472039 | *Priestia aryabhattai* | 99.9 | 1426 | OK474770 |
| AWC24 | 26°C | R2A | OM472040 | *Bacillus* sp. | 100 | 1420 | MT673836 |
| AWC27 | 26°C | R2A | OM472041 | *Bacillus subtilis* | 100 | 1420 | MK286957 |
| AWC31 | 26°C | R2A | OM472042 | *Bacillus licheniformis* | 99.6 | 1414 | JQ236634 |
| AWC36 | 26°C | R2A | OM472043 | *Bacillus* sp. | 99.5 | 1241 | AJ315058 |
| AWC39 | 26°C | LB | OM472044 | *Bacillus subtilis* | 99.9 | 1397 | MW246959 |
| AWC48 | 26°C | LB | OM472045 | *Bacillus sonorensis* | 98.6 | 1324 | KY378902 |
| AWC49 | 26°C | LB | OM472046 | *Peribacillus* sp. | 99.9 | 1425 | OK274121 |
| AWC51 | 26°C | LB | OM472047 | *Bacillus haynesii* | 100 | 1422 | MN704468 |
| AWC53 | 26°C | LB | OM472048 | *Bacillus* sp*.* | 98.4 | 1298 | HM242294 |
| AWC54 | 26°C | LB | OM472049 | *Paenibacillus* sp. | 96.4 | 1187 | MN998546 |
| AWC55 | 26°C | LB | OM472050 | *Bacillus subtilis* | 99.6 | 1397 | MK318244 |
| AWC56 | 26°C | LB | OM472051 | *Peribacillus* sp. | 99.9 | 1425 | OK274121 |
| AWC57 | 26°C | LB | OM472052 | *Bacillus vallismortis* | 100 | 1421 | MF470196 |
| AWC58 | 26°C | LB | OM472053 | *Bacillus cereus* | 100 | 1400 | OK493787 |
| AWC61 | 26°C | R2A 10% NaCl | OM472054 | *Bacillus* sp. | 99.9 | 1423 | MT673790 |
| AWC65 | 26°C | R2A 10% NaCl | OM472055 | *Bacillus* sp. | 99.9 | 1423 | MT673790 |
| AWC68 | 26°C | R2A 3% NaCl | OM472056 | *Peribacillus* sp. | 99.9 | 1422 | OK274121 |
| AWC77 | 26°C | R2A 3% NaCl | OM472057 | *Bacillus subtilis* | 100 | 1414 | OK618379 |
| AWC78 | 26°C | R2A 3% NaCl | OM472058 | *Bacillus paralicheniformis* | 99.3 | 1151 | MT039446 |
| AWC79 | 26°C | R2A 3% NaCl | OM472059 | *Bacillus subtilis* | 99.9 | 1418 | MZ798377 |
| AWC81 | 26°C | R2A 3% NaCl | OM472060 | *Bacillus vallismortis* | 94.7 | 1248 | MN998546 |
| AWC88 | 26°C | R2A | OM472061 | *Bacillus haynesii* | 100 | 1419 | MT789069 |
| AWC89 | 26°C | R2A | OM472062 | *Bacillus paralicheniformis* | 99.7 | 1358 | OU570686 |
| AWC92 | 26°C | R2A | OM472063 | *Bacillus licheniformis* | 99.3 | 1264 | MT538580 |
| AWC99 | 26°C | R2A 1:10 | OM472064 | *Bacillus licheniformis* | 100 | 1397 | MZ453072 |
| AWC100 | 26°C | LB | OM472065 | *Halomonas* sp*.* | 99.9 | 1397 | KC934939 |
| AWR3 | 26°C | R2A | OM472066 | *Bacillus* sp*.* | 99.3 | 1401 | CP063163 |
| AWR4 | 26°C | LB | OM472067 | *Bacillus* sp*.* | 99.9 | 1425 | MW905618 |
| AWR6 | 26°C | R2A 10% NaCl | OM472069 | *Bacillus* sp*.* | 99.9 | 1423 | MT673790 |
| AWR9 | 26°C | R2A 3% NaCl | OM472070 | *Paenibacillus* sp*.* | 98.07 | 1165 | MN998546 |
| AWR10 | 26°C | R2A 3% NaCl | OM472071 | *Paenibacillus* sp*.* | 97.5 | 1146 | KF441657 |
| AWR12 | 26°C | R2A 3% NaCl | OM472072 | *Paenibacillus* sp. | 97.8 | 1155 | MN998546 |
| AWR14 | 26°C | R2A 3% NaCl | OM472073 | *Bacillus licheniformis* | 99.8 | 1424 | KP178603 |
| AWR22 | 55°C | R2A | OM472074 | *Bacillus licheniformis* | 99 | 1208 | KP979476.1 |
| AWR24 | 26°C | R2A | OM472075 | *Bacillus haynesii* | 98.7 | 1290 | NR_157609 |
| AWR31 | 26°C | LB | OM472076 | *Bacillus paralicheniformis* | 100 | 1361 | OU570686 |
| AWR36 | 26°C | LB | OM472077 | *Nafulsella* sp. | 98.2 | 1385 | MH279683 |
| AWR38 | 26°C | R2A 10% NaCl | OM472078 | *Bacillus licheniformis* | 100 | 1421 | MZ453072 |
| AWR39 | 26°C | R2A 10% NaCl | OM472079 | *Bacillus* sp. | 100 | 1416 | MT673836 |
| AWR41 | 26°C | R2A 10% NaCl | OM472080 | *Bacillus haynesii* | 99.9 | 1376 | NR_157609 |
| AWR42 | 26°C | R2A 3% NaCl | OM472081 | *Bacillus licheniformis* | 100 | 1398 | MZ453072 |
| AWR43 | 26°C | R2A 3% NaCl | OM472082 | *Bacillus licheniformis* | 100 | 1415 | MZ453072 |
| AWR44 | 26°C | R2A 3% NaCl | OM472083 | *Bacillus licheniformis* | 100 | 1399 | MZ453072 |
| AWR51 | 26°C | R2A 3% NaCl | OM472084 | *Bacillus licheniformis* | 99.9 | 1416 | MW130990 |
| AWS10 | 55°C | R2A | OM472085 | *Bacillus haynesii* | 99.9 | 1409 | MT789069.1 |
| AWS12 | 26°C | R2A 3% NaCl | OM472086 | *Bacillus haynesii* | 99.9 | 1395 | NR_157609 |
| AWS13 | 26°C | R2A 3% NaCl | OM472087 | *Bacillus haynesii* | 99.9 | 1396 | NR_157609 |
| AWS14 | 26°C | R2A 3% NaCl | OM472088 | *Bacillus haynesii* | 99.9 | 1396 | NR_157609 |

Final species-level assignments for the five strains selected for whole-genome sequencing (AWC2, AWC16, AWC57, AWC81, and AWS14) were confirmed using genome-based taxonomic analysis.

**Supplementary Table 3.** Carbohydrate active enzyme annotations for the five selected strains isolated from Al Wahbah Crater.

|  | **AWC2 *Bacillus spizizenii*** | | **AWC16 *Bacillus cereus*** | | **AWC57 *Bacillus vallismortis*** | | **AWC81 *Bacillus vallismortis*** | | **AWS14 *Bacillus haynesii*** | |
| --- | --- | --- | --- | --- | --- | --- | --- | --- | --- | --- |
| **Class** | **EC^1^** | **Activity** | **EC** | **Activity** | **EC** | **Activity** | **EC** | **Activity** | **EC** | **Activity** |
| Transferases | 2.4.1.1 | glycogen phosphorylase | 2.4.1.1 | glycogen phosphorylase | 2.4.1.1 | glycogen phosphorylase | 2.4.1.1 | glycogen phosphorylase | 2.4.1.1 | glycogen phosphorylase |
|  | 2.4.1.10 | levansucrase | 2.4.1.10 | levansucrase | 2.4.1.10 | levansucrase | 2.4.1.10 | levansucrase | 2.4.1.129 | murein polymerase |
|  | 2.4.1.18 | a-1,4-glucan branching enzyme | 2.4.1.18 | a-1,4-glucan branching enzyme | 2.4.1.18 | a-1,4-glucan branching enzyme | 2.4.1.18 | a-1,4-glucan branching enzyme | 2.4.1.18 | a-1,4-glucan branching enzyme |
|  | 2.4.1.21 | starch glucosyltransferase | 2.4.1.21 | starch glucosyltransferase | 2.4.1.21 | starch glucosyltransferase | 2.4.1.21 | starch glucosyltransferase | 2.4.1.21 | starch glucosyltransferase |
|  | 2.4.1.25 | 4-alpha-glucanotransferase | 2.4.1.25 | 4-alpha-glucanotransferase | 2.4.1.25 | 4-alpha-glucanotransferase | 2.4.1.25 | 4-alpha-glucanotransferase | 2.4.1.25 | 4-alpha-glucanotransferase |
|  | 2.4.1.8 | maltose phosphorylase | 2.4.1.8 | maltose phosphorylase |  |  |  |  |  |  |
| Hydrolases | 3.1.1.6 | acetylesterase | 3.2.1.1 | a-amylase | 3.1.1.72 | acetylxylan esterase | 3.1.1.72 | acetylxylan esterase | 3.2.1.1 | a-amylase |
|  | 3.1.1.72 | acetylxylan esterase | 3.2.1.10 | oligo-a-1,6-glucosidase | 3.2.1.1 | a-amylase | 3.2.1.1 | a-amylase | 3.2.1.10 | oligo-a-1,6-glucosidase |
|  | 3.1.1.73 | feruloyl esterase | 3.2.1.120 | iso primeverose-producing oligo xyloglucan hydrolase | 3.2.1.10 | oligo-a-1,6-glucosidase | 3.2.1.10 | oligo-a-1,6-glucosidase | 3.2.1.132 | chitosanase |
|  | 3.2.1.1 | a-amylase | 3.2.1.122 | maltose-6-phosphate glucosidase | 3.2.1.122 | maltose-6-phosphate glucosidase | 3.2.1.122 | maltose-6-phosphate glucosidase | 3.2.1.133 | maltogenic a-amylase |
|  | 3.2.1.10 | oligo-a-1,6-glucosidase | 3.2.1.132 | chitosanase | 3.2.1.132 | chitosanase | 3.2.1.132 | chitosanase | 3.2.1.135 | neopullulanase |
|  | 3.2.1.122 | maltose-6-phosphate glucosidase | 3.2.1.133 | maltogenic a-amylase | 3.2.1.136 | glucuronoarabinoxylan-specific endo-b-1,4-xylanase | 3.2.1.136 | glucuronoarabinoxylan-specific endo-b-1,4-xylanase | 3.2.1.14 | chitinase |
|  | 3.2.1.132 | chitosanase | 3.2.1.135 | neopullulanase | 3.2.1.153 | fructan β-(2,1)-fructosidase/1-exohydrolase | 3.2.1.153 | fructan β-(2,1)-fructosidase/1-exohydrolase | 3.2.1.17 | lysozyme |
|  | 3.2.1.133 | maltogenic a-amylase | 3.2.1.14 | chitinase | 3.2.1.20 | a-glucosidase | 3.2.1.20 | a-glucosidase | 3.2.1.20 | a-glucosidase |
|  | 3.2.1.135 | neopullulanase | 3.2.1.153 | fructan β-(2,1)-fructosidase/1-exohydrolase | 3.2.1.21 | β-glucosidase | 3.2.1.21 | β-glucosidase | 3.2.1.21 | β-glucosidase |
|  | 3.2.1.153 | fructan β-(2,1)-fructosidase/1-exohydrolase | 3.2.1.17 | lysozyme | 3.2.1.22 | α-galactosidase | 3.2.1.22 | α-galactosidase | 3.2.1.26 | β-fructofuranosidase |
|  | 3.2.1.20 | a-glucosidase | 3.2.1.176 | reducing end-acting cellobiohydrolase | 3.2.1.23 | β-galactosidase | 3.2.1.23 | β-galactosidase | 3.2.1.33 | amylo-alpha-1,6-glucosidase |
|  | 3.2.1.21 | β-glucosidase | 3.2.1.177 | α-xylosidase | 3.2.1.26 | invertase | 3.2.1.26 | β-fructofuranosidase | 3.2.1.4 | endo-b-1,4-glucanase |
|  | 3.2.1.22 | α-galactosidase | 3.2.1.20 | a-glucosidase | 3.2.1.33 | amylo-alpha-1,6-glucosidase | 3.2.1.33 | amylo-alpha-1,6-glucosidase | 3.2.1.41 | pullulanase |
|  | 3.2.1.23 | β-galactosidase | 3.2.1.21 | β-glucosidase | 3.2.1.37 | xylan 1,4-b-xylosidase | 3.2.1.37 | xylan 1,4-b-xylosidase | 3.2.1.54 | cyclo maltodextrin |
|  | 3.2.1.26 | β-fructofuranosidase | 3.2.1.22 | α-galactosidase | 3.2.1.4 | endo-b-1,4-glucanase | 3.2.1.4 | endo-b-1,4-glucanase | 3.2.1.68 | Isoamylase |
|  | 3.2.1.33 | amylo-alpha-1,6-glucosidase | 3.2.1.23 | β-galactosidase | 3.2.1.41 | pullulanase | 3.2.1.41 | pullulanase | 3.2.1.70 | gluco dextranase |
|  | 3.2.1.37 | xylan 1,4-b-xylosidase | 3.2.1.26 | invertase | 3.2.1.52 | β-N-acetylhexosaminidase | 3.2.1.52 | β-N-acetylhexosaminidase | 3.2.1.73 | endo-b-1,3-1,4-glucanase |
|  | 3.2.1.4 | endo-b-1,4-glucanase | 3.2.1.33 | amylo-alpha-1,6-glucosidase | 3.2.1.55 | a-L-arabinofuranosidase | 3.2.1.55 | a-L-arabinofuranosidase | 3.2.1.85 | 6-phospho-β-galactosidase |
|  | 3.2.1.41 | pullulanase | 3.2.1.37 | xylan 1,4-b-xylosidase | 3.2.1.6 | endo-b-1,3(4)-glucanase | 3.2.1.6 | endo-b-1,3(4)-glucanase | 3.2.1.86 | 6-phospho-β-glucosidase |
|  | 3.2.1.52 | β-N-acetylhexosaminidase | 3.2.1.4 | endo-β-1,4-glucanase | 3.2.1.64 | β-2,6-fructan 6-levanbiohydrolase | 3.2.1.64 | β-2,6-fructan 6-levanbiohydrolase | 3.2.1.93 | a,a-trehalose-6-phosphate hydrolase |
|  | 3.2.1.54 | cyclo maltodextrin | 3.2.1.41 | pullulanase | 3.2.1.65 | endo-levanase | 3.2.1.65 | endo-levanase | 3.2.1.98 | maltohexaose-producing a-amylase |
|  | 3.2.1.55 | a-L-arabinofuranosidase | 3.2.1.45 | β-glucosylceramidase | 3.2.1.73 | endo-b-1,3-1,4-glucanase | 3.2.1.73 | endo-b-1,3-1,4-glucanase | 5.4.99.11 | isomaltulose synthase |
|  | 3.2.1.6 | endo-b-1,3(4)-glucanase | 3.2.1.52 | β-N-acetylhexosaminidase | 3.2.1.78 | β-mannanase | 3.2.1.78 | β-mannanase |  |  |
|  | 3.2.1.64 | β-2,6-fructan 6-levanbiohydrolase | 3.2.1.54 | cyclo maltodextrin | 3.2.1.8 | endo-1,4-beta-xylanase | 3.2.1.8 | endo-β-1,4-xylanase |  |  |
|  | 3.2.1.65 | endo-levanase | 3.2.1.55 | a-L-arabinofuranosidase | 3.2.1.80 | exo-inulinase | 3.2.1.80 | exo-inulinase |  |  |
|  | 3.2.1.70 | gluco dextranase | 3.2.1.6 | endo-b-1,3(4)-glucanase | 3.2.1.85 | 6-phospho-β-galactosidase | 3.2.1.85 | 6-phospho-β-galactosidase |  |  |
|  | 3.2.1.73 | endo-b-1,3-1,4-glucanase | 3.2.1.64 | β-2,6-fructan 6-levanbiohydrolase | 3.2.1.86 | 6-phospho-β-glucosidase | 3.2.1.86 | 6-phospho-β-glucosidase |  |  |
|  | 3.2.1.78 | β-mannanase | 3.2.1.65 | endo-levanase | 3.2.1.93 | a,a-trehalose-6-phosphate hydrolase | 3.2.1.93 | a,a-trehalose-6-phosphate hydrolase |  |  |
|  | 3.2.1.8 | endo-1,4-beta-xylanase | 3.2.1.70 | gluco dextranase | 3.2.1.99 | endo-a-1,5-L-arabinanase | 3.2.1.99 | endo-a-1,5-L-arabinanase |  |  |
|  | 3.2.1.80 | exo-inulinase | 3.2.1.73 | licheninase |  |  |  |  |  |  |
|  | 3.2.1.85 | 6-phospho-β-galactosidase | 3.2.1.74 | glucan 1,4-β-glucosidase |  |  |  |  |  |  |
|  | 3.2.1.86 | 6-phospho-β-glucosidase | 3.2.1.78 | β-mannanase |  |  |  |  |  |  |
|  | 3.2.1.89 | endo-β-1,4-galactanase | 3.2.1.8 | endo-β-1,4-xylanase |  |  |  |  |  |  |
|  | 3.2.1.93 | a,a-trehalose-6-phosphate hydrolase | 3.2.1.80 | exo-inulinase |  |  |  |  |  |  |
|  | 3.2.1.99 | endo-a-1,5-L-arabinanase | 3.2.1.85 | 6-phospho-β-galactosidase |  |  |  |  |  |  |
|  |  |  | 3.2.1.86 | 6-phospho-β-glucosidase |  |  |  |  |  |  |
|  |  |  | 3.2.1.89 | endo-β-1,4-galactanase |  |  |  |  |  |  |
|  |  |  | 3.2.1.91 | cellobiohydrolase |  |  |  |  |  |  |
|  |  |  | 3.2.1.93 | a,a-trehalose-6-phosphate hydrolase |  |  |  |  |  |  |
|  |  |  | 3.2.1.98 | maltohexaose-producing a-amylase |  |  |  |  |  |  |
|  |  |  | 3.2.1.99 | endo-a-1,5-L-arabinanase |  |  |  |  |  |  |
|  |  |  |  |  |  |  |  |  |  |  |
| Lyases | 4.2.2.2 | pectate lyase | 4.2.2.2 | pectate lyase | 4.2.2.2 | pectate lyase | 4.2.2.2 | pectate lyase |  |  |
|  | 4.2.2.23 | rhamnogalacturonan endolyase | 4.2.2.23 | rhamnogalacturonan endolyase | 4.2.2.23 | rhamnogalacturonan endolyase | 4.2.2.23 | rhamnogalacturonan endolyase |  |  |
|  |  |  | 4.2.2.9 | exo-polygalacturonate lyase |  |  |  |  |  |  |
| Isomerases | 5.4.99.11 | isomaltulose synthase | 5.4.99.11 | isomaltulose synthase |  |  |  |  |  |  |

^1^The EC columns present the identification number in the Enzyme Commission numbering system for each enzyme.

**Supplementary Table 4.** Annotations of secondary metabolite biosynthesis gene clusters for the selected strains isolated from Al Wahbah Crater.

|  | Type | Most similar known cluster | Similarity |
| --- | --- | --- | --- |
| AWC2 *Bacillus spizizenii* | NRPS, transAT-PKS, T3PKS, PKS-like | Bacillaene | 100% |
|  | NRPS | Bacillibactin | 100% |
|  | TransAT-PKS, NRPS, beta lactone | Mycosubtilin | 100% |
|  | Lanthipeptide-class-i | Subtilin | 100% |
|  | Other, sactipeptide | Subtilosin A | 100% |
|  | NRPS | Surfactin | 82% |
|  | T3PKS |  |  |
|  | CDPS |  |  |
|  | Terpene |  |  |
|  | Terpene |  |  |
| AWC16 *Bacillus cereus* | NRPS | bacillibactin | 46% |
|  | NRPS | bacitracin | 55% |
|  | NRPS | bacitracin | 33% |
|  | NRPS | bacitracin | 33% |
|  | Beta lactone | fengycin | 40% |
|  | Terpene | molybdenum cofactor | 17% |
|  | Siderophore | petrobactin | 100% |
|  | Ranthipeptide |  |  |
|  | LAP, RiPP-like |  |  |
|  | Lassopeptide |  |  |
|  | NRPS |  |  |
|  | RiPP-like |  |  |
|  | NRPS-like |  |  |
|  | NRPS |  |  |
|  | NRPS |  |  |
|  | NRPS,transAT-PKS, NRPS-like |  |  |
|  | NRPS |  |  |
| AWC57 *Bacillus vallismortis* | NRPS, RiPP-like | bacillibactin | 100% |
|  | Betalactone | fengycin | 66% |
|  | Lanthipeptide-class-i | subtilin | 100% |
|  | Sactipeptide | subtilosin A | 37% |
|  | NRPS | surfactin | 82% |
|  | NRPS, T1PKS | zwittermicin A | 18% |
|  | terpene |  |  |
|  | TransAT-PKS-like |  |  |
|  | CDPS |  |  |
|  | T3PKS |  |  |
|  | Terpene |  |  |
|  | NRPS |  |  |
| AWC81 *Bacillus vallismortis* | NRPS, transAT-PKS, T3PKS, PKS-like | bacillaene | 92% |
|  | NRPS, RiPP-like | bacillibactin | 100% |
|  | Other | bacilysin | 100% |
|  | Lanthipeptide-class-i | entianin | 36% |
|  | NRPS,betalactone | fengycin | 86% |
|  | NRPS | plipastatin | 30% |
|  | NRPS | plipastatin | 23% |
|  | Sactipeptide | subtilosin A | 25% |
|  | NRPS | surfactin | 82% |
|  | Terpene |  |  |
|  | Terpene |  |  |
|  | T3PKS |  |  |
|  | Epipeptide |  |  |
|  | CDPS |  |  |
| AWS14 *Bacillus haynesii* | NRPS | bacillibactin | 53% |
|  | NRPS | bacitracin | 100% |
|  | Thiopeptide, RiPP-like | butirosin A / butirosin B | 7% |
|  | NRPS, betalactone | fengycin | 86% |
|  | NRPS | lichenysin | 57% |
|  | NRPS | lichenysin | 50% |
|  | NRPS | lichenysin | 14% |
|  | Terpene |  |  |
|  | T3PKS |  |  |
|  | CDPS |  |  |
|  | Lassopeptide |  |  |
|  | RiPP-like |  |  |
|  | Siderophore |  |  |
|  | Thiopeptide |  |  |

**Supplementary Table 5.** Predicted identities of the prophage regions found in the selected strains isolated from Al Wahbah Crater.

|  | Region length | Completeness | Most common phage |
| --- | --- | --- | --- |
| AWC2 *Bacillus spizizenii* | 11.1 Kb | Incomplete | PHAGE_Bacill_SPbeta_NC_001884(2) |
|  | 9.3 Kb | Incomplete | PHAGE_Bacill_phiNIT1_NC_021856(2) |
|  | 20.6 Kb | Incomplete | PHAGE_Brevib_Davies_NC_022980(4) |
|  | 20.3 Kb | Incomplete | PHAGE_Brevib_Jimmer1_NC_029104(5) |
| AWC16 *Bacillus cereus* | 10.9 Kb | Incomplete | PHAGE_Bacill_phi4J1_NC_029008(3) |
|  | 18.6 Kb | Questionable | PHAGE_Bacill_IEBH_NC_011167(7) |
|  | 15.3 Kb | Incomplete | PHAGE_Bacill_Waukesha92_NC_025424(2) |
|  | 35.3 Kb | Intact | PHAGE_Lister_2389_NC_003291(15) |
|  | 13.4 Kb | Incomplete | PHAGE_Bacill_phBC6A52_NC_004821(8) |
| AWC57 *Bacillus vallismortis* | 34.1 Kb | Incomplete | PHAGE_Brevib_Jimmer2_NC_041976(9) |
|  | 5.5 Kb | Incomplete | PHAGE_Bacill_SPbeta_NC_001884(2) |
|  | 9.1 Kb | Incomplete | PHAGE_Bacill_SPbeta_NC_001884(9) |
| AWC81 *Bacillus vallismortis* | 11.7 Kb | Incomplete | PHAGE_Brevib_Osiris_NC_028969(2) |
|  | 30.1 Kb | Incomplete | PHAGE_Clostr_phiCT453A_NC_028991(7) |
|  | 35.1 Kb | Intact | PHAGE_Brevib_Jimmer2_NC_041976(8) |
|  | 19.6 Kb | Incomplete | PHAGE_Staphy_SPbeta_like_NC_029119(2) |
|  | 25.1 Kb | Incomplete | PHAGE_Bacill_SPbeta_NC_001884(5) |
| AWS14 *Bacillus haynesii* | 38.3 Kb | Incomplete | PHAGE_Bacill_SPbeta_NC_001884(2) |
|  | 34.5 Kb | Intact | PHAGE_Brevib_Jimmer2_NC_041976(7) |
|  | 42.9 Kb | Questionable | PHAGE_Bacill_phi105_NC_004167(21) |
|  | 70.1 Kb | Intact | PHAGE_Paenib_Tripp_NC_028930(28) |
|  | 20.3 Kb | Incomplete | PHAGE_Clostr_phiCD505_NC_028764(2) |
|  | 12.9 Kb | Incomplete | PHAGE_Bacill_phi105_NC_048631(4) |
